# The Roles of Cortical Slow Waves in Synaptic Plasticity and Memory Consolidation

**DOI:** 10.3389/fncir.2017.00092

**Published:** 2017-11-22

**Authors:** Daisuke Miyamoto, Daichi Hirai, Masanori Murayama

**Affiliations:** ^1^Laboratory for Behavioral Neurophysiology, RIKEN Brain Science Institute, Wako, Japan; ^2^Japan Society for the Promotion of Science (JSPS), Tokyo, Japan

**Keywords:** cortex, hippocampus, memory, oscillation, sleep, synaptic plasticity

## Abstract

Sleep plays important roles in sensory and motor memory consolidation. Sleep oscillations, reflecting neural population activity, involve the reactivation of learning-related neurons and regulate synaptic strength and, thereby affect memory consolidation. Among sleep oscillations, slow waves (0.5–4 Hz) are closely associated with memory consolidation. For example, slow-wave power is regulated in an experience-dependent manner and correlates with acquired memory. Furthermore, manipulating slow waves can enhance or impair memory consolidation. During slow wave sleep, inter-areal interactions between the cortex and hippocampus (HC) have been proposed to consolidate declarative memory; however, interactions for non-declarative (HC-independent) memory remain largely uninvestigated. We recently showed that the directional influence in a slow-wave range through a top-down cortical long-range circuit is involved in the consolidation of non-declarative memory. At the synaptic level, the average cortical synaptic strength is known to be potentiated during wakefulness and depressed during sleep. Moreover, learning causes plasticity in a subset of synapses, allocating memory to them. Sleep may help to differentiate synaptic strength between allocated and non-allocated synapses (i.e., improving the signal-to-noise ratio, which may facilitate memory consolidation). Herein, we offer perspectives on inter-areal interactions and synaptic plasticity for memory consolidation during sleep.

## Introduction

Neural oscillations during sleep support memory consolidation, which refers to post-learning processes whereby new memory transitions from a fragile to a stable form. The existence of the memory consolidation process has been verified by the transient susceptibility of memory to amnesic interventions including sleep-deprivation and drugs inactivating neural activity or preventing synaptic plasticity (Dudai et al., 2015). Synchronized neural activity during sleep forms oscillations with distinct frequencies. During non-rapid eye movement (NREM) sleep, three major oscillations are involved in memory consolidation: slow waves that originate from the neocortex; 12–15 Hz spindles that arise from the thalamus and spread to cortical and hippocampal networks (Lustenberger et al., 2016); and 140–200 Hz hippocampal ripples (Girardeau et al., 2009; Ego-Stengel and Wilson, 2010; Maingret et al., 2016). Slow waves reflect spontaneous alterations of depolarized (up) and hyperpolarized (down) states in cortical neurons (Vyazovskiy and Harris, 2013). They typically originate from layer 5 neurons and spread to other cortical layers (Chauvette et al., 2010). Among cortical areas, slow waves can occur locally, but become more global during deep sleep (Nir et al., 2011; Vyazovskiy et al., 2011). Although slow waves can spread over cortical areas as traveling waves that may originate from different sites, they typically originate in the frontal cortex (FC) in humans (Massimini et al., 2004). While slow waves are prominent during deep NREM sleep, spindles are more obvious during light NREM sleep (Steriade, 2003). Spindles are generated through the interplay of GABAergic interneurons of the reticular nucleus of the thalamus and thalamocortical cells (Steriade et al., 1985, 1987; Halassa et al., 2011). However, corticothalamic projections govern the widespread synchronization of spindles among thalamic areas (Contreras and Steriade, 1996; Contreras et al., 1996; Steriade, 2003). Ripples in the hippocampus (HC) are synchronized with cortical up-states and spindles (Siapas and Wilson, 1998; Sirota et al., 2003; Rothschild et al., 2017), and have been proposed to operate in the inter-areal interactions between the HC and cortex in memory consolidation. During rapid eye movement (REM) sleep, memory consolidation is dependent on medial septum gamma-aminobutyric acid (GABA)-releasing neurons, which generates 4–10 Hz theta oscillations in the HC (Boyce et al., 2016). Among these sleep oscillations, slow waves have significant roles in memory consolidation, which has clinical implications for enhancing memory, because triggering slow waves in humans and animals can enhance memory consolidation (Marshall et al., 2006; Binder et al., 2013, 2014; Ngo et al., 2013, 2015; Miyamoto et al., 2016; Rembado et al., 2017).

Sleep also has functional significance in synaptic plasticity, which is a well-known mechanism involved in learning and memory. Synaptic plasticity has several activity-dependent rules. In the Bienenstock-Cooper-Munro (BCM) frequency-dependent curve (Bienenstock et al., 1982; Cooper and Bear, 2012; Jedlicka et al., 2015), pre-synaptic stimulation at low frequency (~1 Hz) causes synaptic depression, while stimulation at high frequency (>10 Hz) causes synaptic potentiation. Thus, slow waves are believed to depress synaptic activity, although recent studies have shown that slow-wave-like stimulation or lower-frequency (0.1 Hz) stimulation can cause synaptic potentiation (Chauvette et al., 2012; Sandler et al., 2016). In the spike-timing-dependent plasticity (STDP) rule (Dan and Poo, 2004), spike or activity order in pre-synapse and post-synapse define the direction of synaptic plasticity. Hierarchical top-down slow-wave flow (Massimini et al., 2004; Nir et al., 2011; Miyamoto et al., 2016) may lead to synaptic plasticity according to the STDP rule. Furthermore, the hypothesis of synaptic memory allocation, the process that determines which synapses work for specific memory traces—suggests that memory is encoded by a specific subset of synapses (Rogerson et al., 2014; Hayashi-Takagi et al., 2015). During up-states of slow waves, reactivation of neurons (Pavlides and Winson, 1989; Miyamoto et al., 2016) or neuronal ensembles (Ji and Wilson, 2007; Rothschild et al., 2017), which are activated during the awake experience, may differentiate synaptic strength between memory-allocated synapses and non-allocated synapses. Herein, we discuss synaptic plasticity with slow waves representing a candidate mechanism of memory consolidation.

## Slow Waves and Memory Consolidation

### Experience-Dependent Slow Waves

Sensory or motor experience regulates the spectral power of slow waves (slow-wave power) in experience-related brain areas. In humans, tactile stimulation for 6 h in the right hand causes a local increase in slow-wave power in the left hemisphere (Kattler et al., 1994). Moreover, motor learning for several tens of minutes also enhances slow-wave power in the motor area, during post-learning NREM sleep, and this increase in slow-wave power correlates with motor performance improvement (Huber et al., 2004). Conversely, arm immobilization for 12 h impairs motor performance and slow-wave power in the sensorimotor area (Huber et al., 2006). Similarly, depriving visual information by means of 3 months dark-rearing reduces slow-wave power in the visual cortex, but not in the somatosensory cortex in cats and mice (Miyamoto et al., 2003). These experience-dependent changes in slow-wave power indicate a correlation between slow waves and memory.

### Causal Roles of Sleep Oscillations in Memory Consolidation

Researchers have manipulated sleep oscillations to demonstrate causal relations to the consolidation of declarative and non-declarative memories (Stickgold, 2005). Non-declarative (HC-independent) memory, which should not be affected by hippocampal ripples, may have different consolidation mechanisms during sleep; however, these mechanisms remain unclear. In humans, boosting slow waves by means of transcranial direct current stimulation (tDCS) in the FC (Marshall et al., 2006) or with closed-loop in-phase auditory stimulation (Ngo et al., 2013) during NREM sleep can enhance declarative memory consolidation in paired-associate (word-pairs) learning tasks. In non-declarative finger-sequence tapping task, boosting spindles enhanced skill memory consolidation (Lustenberger et al., 2016), and closed-loop auditory stimulation, to perturb slow waves in the motor cortex, made motor execution less efficient (Fattinger et al., 2017). Regarding non-declarative (HC-independent) perceptual memory, we have developed a sleep-dependent tactile discrimination task in mice, and applied optogenetic stimulation (Miyamoto and Murayama, 2016) to the task-associated primary somatosensory cortex (S1) and/or secondary motor cortex (M2), which forms a somatosensory perception-related top-down cortical circuit (Manita et al., 2015, 2017). When the stimulation was synchronized with the frequency of slow waves, it resulted in enhanced memory consolidation. Therefore, slow waves can contribute not only to declarative memory but also to non-declarative skill and perceptual memory.

**Figure 1:**
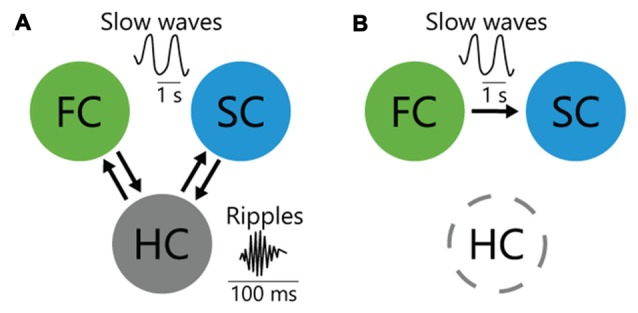
Interareal interactions among cortices and hippocampus (HC) for memory consolidation. **(A)** HC bidirectionally interacts with frontal cortex (FC) and sensory cortex (SC) through entorhinal cortex during sleep, which may contribute to consolidation of HC-dependent memory. **(B)** In HC-independent perceptual memory, top-down inputs from the FC to SC during sleep consolidate memory.

The causal roles of hippocampal ripples in memory consolidation have also been intensively studied. Ripples are believed to mediate transference of reactivated memory information from the HC to the cortex to support systems memory consolidation (i.e., the post-learning, time-dependent reorganization of long-term memory representations over distributed brain areas; Takehara-Nishiuchi et al., 2006; Inostroza and Born, 2013; Dudai et al., 2015; Kitamura et al., 2017). In support of this concept, disruption of ripples with electrical stimulation impaired consolidation of HC-dependent spatial memory (Girardeau et al., 2009; Ego-Stengel and Wilson, 2010). To more directly test the causal effect of the hippocampo-cortical dialog on memory consolidation, Maingret et al. (2016) enhanced the temporal correlation between the HC and medial prefrontal cortex using ripple-triggered electrical stimulation of deep cortical layers (Maingret et al., 2016). Because triggering cortical oscillations after hippocampal ripples enhanced spatial memory performance, information flow from the HC to the cortex is believed to be important for memory consolidation. Conversely, information flow from the prefrontal cortex or sensory cortex (SC) to the HC (Isomura et al., 2006; Rothschild et al., 2017) may also function in memory consolidation (Figure 1A). Whether bidirectional or unidirectional interaction between the HC and cortex is involved in memory consolidation is yet to be determined.

Inter-areal interactions have been studied extensively in HC-dependent declarative memory; however, those for HC-independent non-declarative memory remain unclear (Dudai et al., 2015). To elucidate the inter-areal interactions for non-declarative tactile-perceptual memory task, we optogenetically manipulated a bidirectional cortico-cortical pathway between anterior M2 and posterior S1, which supports tactile perception (Manita et al., 2015). While suppressing the posteroanterior S1→M2 (i.e., bottom-up) pathway did not impair memory consolidation, suppressing the anteroposterior M2→S1 (i.e., top-down) slow-wave flow impaired emergence of experience-related reactivated neuron in S1 and impaired memory consolidation, which initially demonstrated the causal contribution of the top-down pathway (Figure 1B). Because similar hierarchical slow-wave flows from the anterior to the posterior cortex during NREM sleep are observed globally in the cortex (Massimini et al., 2004; Nir et al., 2011; Phillips et al., 2012), they may also function in memory reactivation and consolidation in other modalities.

**Figure 2:**
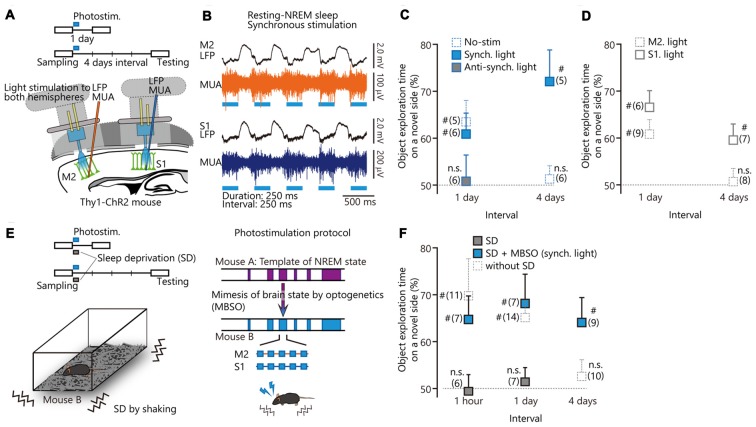
Optogenetic cortical stimulation in slow-wave rhythm for enhancing or impairing memory consolidation.** (A)** Optogenetic activation of secondary motor cortex (M2) and primary somatosensory cortex (S1) with local field potential (LFP) recordings using single electrodes. See “Methods” in Miyamoto et al. (2016). **(B)** LFPs and multiunit activity (MUA) recordings from M2 (top) and S1 (bottom) during resting-non-rapid eye movement (NREM) sleep with synchronous photostimulation. **(C,D)** Task performance after photostimulation during resting-NREM sleep. **(E)** (Left) Behavioral paradigm. After the sampling period, transgenic mice were deprived of sleep for 1 h with synchronized coactivation of M2 and S1. (Right) Photostimulation protocol. The photostimulation (2 Hz) during the sleep deprivation (SD) experiment was applied according to the NREM sleep pattern from mouse A. **(F)** Summary of task performance after photostimulation. The cumulative illumination time was 30 min. Statistical significance from 50% chance level (^#^*P* < 0.05) was assessed by a 1-sample *t* test. Panels **(A–C,E)** and a part of **(F)** from Miyamoto et al. (2016). Reprinted with permission from AAAS.

To test the sufficiency of slow waves to elicit memory consolidation, we optogenetically stimulated layer 5 neurons to trigger slow wave production, as has been done previously (Beltramo et al., 2013). In Figure 2, we reproduced from our previous study (Miyamoto et al., 2016) and added new data in Figure 2D and a part of Figure 2F. By using optogenetic stimulation during NREM sleep, we synchronized or anti-synchronized slow waves between M2 and S1 (Miyamoto et al., 2016). Synchronized stimulation enhanced the retention periods of tactile recognition memory, while anti-synchronized stimulation impaired memory consolidation (Figure 2C from Miyamoto et al., 2016). A recent optogenetic study reported that neuronal activity in the visual cortex during NREM sleep was required for visual experience-dependent plasticity (Durkin et al., 2017). In reverse, to reveal the brain area that is sufficient for enhancement of perceptual memory consolidation, we applied optogenetic stimulation to S1 or M2, alone. Stimulation of M2 *per se* did not enhance tactile memory retention periods (Figure 2D). The stimulation of S1 *per se* could enhance memory retention periods for at least 4 days, indicating that S1 is sufficient for memory consolidation (Figure 2D). However, mouse performance with the stimulation (Figure 2D) was lower than that with the synchronized stimulation of S1 and M2 (Figure 2C from Miyamoto et al., 2016). These results indicate that coordinated activation of S1 and M2 via top-down slow wave flow is more beneficial for memory consolidation than activation of the sensory area alone. Nonetheless, selective stimulation of a sensory area may have advantages in terms of selective enhancement of memory consolidation in one modality, without affecting systems involving other modalities.

To test whether memory consolidation is supported by slow waves *per se* or by slow waves in the context of global cortical activity and neuromodulation during sleep, we used optogenetic stimulation in sleep-deprived mice. We stimulated M2 and S1 synchronously at 2 Hz to mimic sleep slow waves during sleep-deprived states (Figure 2E from Miyamoto et al., 2016). This synchronous stimulation not only restored memory deficits during sleep deprivation (SD), but prolonged memory retention periods compared to mice with normal sleep (Figure 2F from Miyamoto et al., 2016, excepting data sets of 1 h and 4 days, without SD). Memory consolidation during awake and sleep periods is not only a fundamental question in neuroscience but may also have implications for the treatment of memory deficits in patients with sleep disorders such as insomnia (Backhaus et al., 2006).

We demonstrated that artificial optogenetic stimulation of layer 5 neurons in slow-wave rhythm could restore deficits in memory consolidation caused by SD. This, however, leads to an important question: is it possible to consolidate memory only with spontaneous NREM-like cortical slow waves during REM sleep (Funk et al., 2016) or during active wakefulness (Fisher et al., 2016)? In our perceptual memory task, memory consolidation was dependent on sleep 0–1 h after learning. The 1 h includes the total duration of wakefulness (approximately 43%), NREM (approximately 55%) and REM (approximately 3%; Miyamoto et al., 2016). Because the REM state period was negligible, and spontaneous slow waves are rare and not repetitive during active wakefulness (Vyazovskiy and Harris, 2013; Fisher et al., 2016), neural activity in slow-wave rhythm in the NREM periods, when we applied optogenetic stimulation to sleep-deprived awake mice, would be important for memory consolidation.

## Slow Waves and Synaptic Plasticity

### Slow Waves in Synaptic Depression and Potentiation

The sleep synaptic homeostasis hypothesis (SHY), a leading hypothesis of synaptic plasticity through the sleep/wake cycle, states that the net synaptic strength increases during wakefulness and decreases during sleep (Tononi and Cirelli, 2003, 2006, 2014; Born and Feld, 2012). Slow waves may be related to sleep synaptic depression according to the BCM synaptic modification rule (i.e., low-frequency stimulation induces long-term depression), which was theorized to account for experience-dependent modification of neural activity in the visual cortex (Bienenstock et al., 1982; Nakao et al., 2004; Cooper and Bear, 2012). In fact, the SHY is supported by molecular evidence: protein expression levels of GluA1-containing AMPA receptors, glutamate receptors mediating excitatory inputs, are higher during the active phase in the animal’s circadian rhythm than during the inactive phase, in the cortex and the HC (Vyazovskiy et al., 2008). The SHY is also supported by structural evidence: the net spine number and the size of axon-spine interface (ASI) decreases after sleep (Maret et al., 2011; de Vivo et al., 2017). Moreover, synaptic efficacy measured electrophysiologically in cortico-cortical synapses increases after wake and decreases after sleep (Vyazovskiy et al., 2008). Although these studies have shown synaptic plasticity on average, they support the SHY in the sense that net synaptic potentiation occurs during wakefulness and net synaptic depression occurs during sleep in the cortex.

In contrast, some studies have reported that slow waves may induce synaptic potentiation. Chauvette et al. (2012) have shown that synaptic potentiation occurs through sleep in the thalamo-cortical system *in vivo*. They have also shown that recapitulation of slow waves *in vitro* by means of paired presynaptic stimulation and postsynaptic hyperpolarization induces long-term potentiation in layer II/III pyramidal neurons. Surprisingly, unpaired stimulation at even lower frequency (0.1 Hz), which is conventionally used for assessing baseline synaptic strength in many LTP/LTD studies (Urba et al., 1991; Magee and Johnston, 1997; Markram et al., 1997; Sjöström et al., 2001; Gordon et al., 2006), can also induce synaptic potentiation in tuft dendrites (Sandler et al., 2016). The results of these studies are in contradiction to the SHY in the sense that sleep slow wave or even lower-frequency stimulation can induce synaptic potentiation in some pathways.

To summarize the above, some studies are inconsistent with the SHY, which may be due to detection of synaptic potentiation in certain pathways; however, the total synaptic strength assessed by means of the AMPA receptor protein in the cortex is depressed during sleep (Vyazovskiy et al., 2008; Diering et al., 2017).

### Plasticity through Sleep in Individual Neurons and Synapses

Learning activates a subset of neurons, which likely contribute to the memory traces or memory engrams, which are a theoretical means by which memory is physically stored in the brain (Han et al., 2007; Reijmers et al., 2007; Liu et al., 2012; Tonegawa et al., 2015; Rashid et al., 2016). Similarly, only a limited number of neurons increase their firing rates during post-learning sleep as compared to pre-learning sleep, which may be important for memory consolidation (Grosmark and Buzsáki, 2016; Miyamoto et al., 2016). Thus, in the context of memory, plasticity should be considered separately for each neuron. While the total firing rates in the cortex decreases during sleep (Vyazovskiy et al., 2009), the plasticity of each neuron during sleep is not clear. Recent studies in the cortex (Watson et al., 2016) and HC (Miyawaki and Diba, 2016) confirmed that the total firing rates is depressed during sleep; however, the direction of the changes in firing rate differed, depending on the initial firing rate of each neuron. Watson et al. (2016) showed that, during sleep, cortical neurons with initially higher firing rates decreased their firing rates, while neurons with initially lower firing rates increased their firing rates. Watson et al. (2016) suggested that sleep helps to equalize neurons with higher and lower firing rates, and hypothesized that “homeostatic downscaling affects mainly the minority high-firing neurons to provide network stability, whereas ‘silent’ and slow-firing neurons comprise a large pool of reserve for learning, development and regeneration-induced specific plasticity”. To reveal the mechanisms of memory consolidation during sleep, how subsets of neurons related to learning and memory change their firing rates during sleep should be an interesting future question.

Memory traces can also be allocated to a subset of synapses. Motor learning activates and potentiates a subset of synapses in the motor cortex (Yang et al., 2014; Cichon and Gan, 2015), and selective shrinkage of those synapses using optogenetics erases motor memory (i.e., motor skills; Hayashi-Takagi et al., 2015). How does sleep rearrange the synaptic strength of potentiated (allocated) and non-potentiated (non-allocated) synapses for memory consolidation? According to the SHY, two candidate patterns of the plasticity have been suggested (Tononi and Cirelli, 2006, 2014). First, in the global downscaling model, all synapses are depressed non-selectively in proportion to their baseline strength. If the relative relationship in synaptic strength between potentiated and non-potentiated synapses is maintained through synaptic depression during sleep, it may help to prevent deterioration of memory information. Global downscaling is supported by *in vitro* homeostatic plasticity studies in which neural activity level was up- or downregulated globally and markedly (Turrigiano et al., 1998; Davis, 2006; Turrigiano, 2011). Second, the down-selection model implies selectivity, i.e., that some synapses are depressed, while others do not show synaptic depression or show only relatively mild depression. If sleep suppresses non-potentiated, but not potentiated, synapses during learning, the signal-to-noise ratio will increase, which may be good for memory consolidation.

Although these hypotheses have not been tested directly, recent studies (Diering et al., 2017; de Vivo et al., 2017) have shown cortical synaptic depression during sleep, at single-synapse resolution. de Vivo et al. (2017) measured the size of the ASI, the direct area of apposition between the pre- and post-synapse, as an index of synaptic strength, using electron microscopy in mice experiencing sleep or wakefulness (spontaneous wake or SD). At the population level, 80% of the synapses were weaker in the sleep group, while the distribution of the top 20% was similar between the two groups. However, because they could not follow the same synapses through the sleep/wake cycle, it could not be determined how individual synapses change. Diering et al. (2017) used fluorescence-tagged AMPA receptors in dendritic spines and measured their fluorescence intensity to assess synaptic strength at a single-synapse resolution, using *in vivo* time-lapse 2-photon imaging. They showed that synapses with a higher intensity of fluorescence are depressed, while synapses that are less fluorescent are preserved. Cirelli (2017) stated that: “This finding contrasts with the ASI changes described above, which seem to spare the largest spines, but the two studies are difficult to compare directly: they both focused on superficial layers of motor cortex, but the ASI experiments were performed in young mice in which all synapses within the reconstructed dendritic branches were measured, while the two-photon experiment used mature mice and focused on stable spines of the apical dendrites of layer V neurons”. While recent studies have reported sleep-related synaptic depression at a single-synapse resolution, how potentiated synapses with learning are processed during sleep remains a significant question.

## Conclusion

Characteristic oscillations during NREM sleep (slow waves, spindles and ripples) are causally linked to memory consolidation. In humans, visually acquired declarative memory is slow wave-dependent, while non-declarative motor memory is spindle-dependent (Marshall et al., 2006; Ngo et al., 2013; Lustenberger et al., 2016). Similarly, in rodents, visually acquired declarative memory is dependent on slow waves (Binder et al., 2013, 2014) and ripples (Girardeau et al., 2009; Ego-Stengel and Wilson, 2010; Maingret et al., 2016); however, the causal relation between slow waves and non-declarative memory remain unclear. Our optogenetic study in mice demonstrated that slow waves are causally related to non-declarative memory of tactile-perceptual information (Miyamoto et al., 2016). Taken together, while spindles are causally related to motor skill memory, perceptually acquired memory, whether it is HC-dependent or -independent, may be causally related to slow waves.

Interestingly, our study further demonstrated that memory consolidation of somatosensory information can be enhanced by synchronous stimulation to M2 and S1, even during awake periods under SD. This suggests that memory consolidation does not require sleep *per se*, but rather requires sleep oscillations. Furthermore, this finding may be generalized to declarative and other types of non-declarative memory: if slow waves, spindles, and/or ripples are enhanced by external stimuli during awake periods, it may restore deficits in memory due to SD. In contrast, we showed that anti-synchronous stimulation in slow-wave rhythm to M2 and S1 impaired the tactile-perceptual memory in mice. Such a stimulation pattern to specific brain areas underlying a specific function could be a new strategy for erasing unwanted memories, such as fear memories.

Although slow waves consolidate memory, the neural circuitry mechanism(s) remain unclear. First, the activities of individual neurons or neural ensembles during slow waves that cause synaptic plasticity and memory consolidation remain unclear. Recent advances in all-optical imaging and manipulation of neural activity with cellular resolution (Rickgauer et al., 2014; Szabo et al., 2014; Packer et al., 2015; Miyamoto and Murayama, 2016) may be able to visualize and control reactivation during up-states of slow waves and reveal causal roles in synaptic plasticity and memory consolidation. Second, the direction of synaptic plasticity during sleep is controversial. Artificial low-frequency stimulation has been shown to cause either synaptic depression or potentiation, which may be dependent on the stimulation protocol or pathway. Although the net synaptic strength decreases in the cortex and HC through sleep, all synapses may not be depressed uniformly. Instead, memory reactivation during post-learning sleep may cause synaptic potentiation or prevent synaptic depression in some synapses. How oscillation and memory reactivation during post-leaning sleep rearranges synapses that have been modulated through learning is an intriguing question for future investigations.

## Ethics Statement

This study was carried out in accordance with the recommendations of “the guidelines of RIKEN”. The protocol was approved by “the Animal Investigation Committee of the RIKEN BSI”.

## Author Contributions

DM wrote the manuscript. DH performed experiments and analyzed data. MM supervised the project.

## Conflict of Interest Statement

The authors declare that this study received funding from the KAO corporation. The funder was not involved in the study design or collection, analysis, or interpretation of the data.
